# PET/CT-guided dose-painting versus CT-based intensity modulated radiation therapy in locoregional advanced nasopharyngeal carcinoma

**DOI:** 10.1186/s13014-016-0739-y

**Published:** 2017-01-13

**Authors:** Feng Liu, Xu-ping Xi, Hui Wang, Ya-qian Han, Feng Xiao, Ying Hu, Qian He, Lin Zhang, Qin Xiao, Lin Liu, Le Luo, Yun Li, Yi Mo, Hong-zhi Ma

**Affiliations:** 1 Department of Radiation Oncology, Hunan Cancer Hospital and The Affiliated Cancer Hospital of Xiangya School of Medicine, Central South University, Changsha, Hunan China; 2 Key Laboratory of Translational Radiation Oncology, Hunan Province Department of Radiation Oncology, Hunan Cancer Hospital and The Affiliated Cancer Hospital of Xiangya School of Medicine, Central South University, Changsha, Hunan China

**Keywords:** Nasopharyngeal carcinoma, FDG-PET/CT, Dose painting, Intensity-Modulated Radiation Therapy, Toxicity

## Abstract

**Background:**

The effect of ^18^F-fluorodeoxyglucose positron emission tomography/computed tomography (FDG-PET/CT)-guided dose-painting intensity-modulated radiation therapy (IMRT) in locoregionally advanced nasopharyngeal carcinoma (NPC) is unclear. This study aimed to assess the efficacy and toxicity of such combination.

**Methods:**

From 2012 to 2014, 213 patients with stage III-IVB NPC received chemoradiotherapy by PET/CT-guided DP-IMRT (group A, *n* = 101) or CT-based IMRT (group B, *n* = 112). In group A, subvolume GTVnx-_PET_ (gross tumor volume of nasopharynx in PET images) was defined within GTVnx (gross tumor volume of nasopharynx) as the SUV50%max isocontour; the dose to GTVnx-_PET_ was escalated to DT 75.2 Gy/32 and 77.55 Gy/33 Fx, respectively, for patients with T1-2 and T3-4 disease, respectively. In group B, PGTVnx was irradiated at DT 70.4–72.6 Gy/32–33 Fx in 2.2 Gy per fraction.

**Results:**

Complete response rates were 99.0% (100/101) and 92.9% (104/112) in groups A and B, respectively (*P* = 0.037). Compared with CT-based IMRT, FDG-PET/CT guided DP-IMRT significantly improved 3-year local failure-free survival (LFFS, 98.8% vs. 91.3%; *P* = 0.032), locoregional failure-free survival (LRFFS, 97.2 vs. 91.2%; *P* = 0.049), distant metastasis-free survival (DMFS, 92.9% vs. 87.4%; *P* = 0.041), disease free survival (DFS, 87.9% vs. 82.4%; *P* = 0.02), and overall survival (OS, 91.8% vs. 82.6%; *P* = 0.049). No statistically significant differences in acute and late toxic effects were observed. Multivariate analysis showed that dose painting (PET/CT-guided DP-IMRT vs CT-based IMRT without DP) was a significant independent prognostic factor for LFFS and DFS.

**Conclusion:**

FDG-PET/CT guided DP-IMRT plus chemotherapy is associated with a considerable survival benefit, without increasing toxicity in patients with locoregional advanced NPC. Further randomized trials are needed to fully assess the role of PET/CT-guided DP-IMRT.

## Background

Nasopharyngeal carcinoma (NPC) is endemic in Southern China and Southeast Asia [1, 2]; definitive radiotherapy (RT) remains the mainstay of treatment for non-disseminated NPC [1, 3]. Although concurrent chemoradiotherapy is widely used in patients with locally advanced NPC, treatment results remain disappointing. The rate of local residual NPC is about 10%, while that of locally recurrent disease ranges from 16.8 to 23%, depending on initial tumor status [4, 5]. Since local control is directly related to mortality in NPC, there is a strong need to identify methods that further improve treatment outcome in NPC. Escalating the radiotherapy dose could improve local control, which is directly related to radiotherapy dose [6]. Various methods have been used to increase radiotherapy dose, including intracavitary brachytherapy [7, 8], stereotactic radiotherapy [4], and functional image-guided dose-escalation [9]. However, dose escalation in NPC may increase therapy-related complications because of high doses of irradiation to normal tissues [10]. The main challenge for such treatment is therefore to identify the appropriate tumor volume to be prescribed high radiation dose. In NPC, potential advantages of ^18^F-fluorodeoxyglucose positron emission tomography/computed tomography (FDG-PET/CT) are increasingly recognized. PET provides biologic information regarding the tumor, complementing anatomic imaging. ^18^F-FDG-PET/CT possibly promotes accurate delineation and treatment of the tumor and extensions, while reducing the dose received by surrounding normal tissues [11]. We hypothesized that use of PET/CT-guided dose painting (DP) by numbers can determine the appropriate tumor volume to receive high-dose radiotherapy and improve dose-escalation radiotherapy for NPC; this in turn can improve therapeutic efficacy while reducing toxicity. There are limited studies assessing PET/CT guided dose-escalation chemoradiotherapy in locoregionally advanced NPC, with very small sample size [9]. In addition, no clinical trials have directly compared PET/CT-guided dose-painting intensity modulated radiation therapy (DP-IMRT) to CT-based IMRT in locoregionally advanced NPC. Here, we retrospectively compared preliminary efficacy and toxicity profile of PET/CT-guided DP-IMRT versus CT-based IMRT in locoregionally advanced NPC patients.

## Methods

### Eligibility criteria

Between January 2012 and July 2014, 213 patients with locoregional advanced NPC were included in this study. Eligible patients were aged 18–70 years with non-metastatic, histologically proven WHO types II-III, stage III-IVB nasopharyngeal carcinoma (7^th^ American Joint Committee on Cancer, AJCC). All participants had Karnofsky scores of at least 70, and adequate bone marrow (leukocyte count ≥4.0 × 10^9^/L, absolute neutrophil count ≥1.5 × 10^9^/L, platelets ≥100 × 10^9^/L), liver, and renal functions. Patients were required to provide written informed consent before treatment. Exclusion criteria were: previous radiotherapy, another malignancy, pregnancy or lactation, active infection or unstable cardiac disease needing treatment. All patients were recruited from Hunan Cancer Hospital at The Affiliated Cancer Hospital of Xiangya School of Medicine, Central South University.

### Radiotherapy

All patients underwent a pretreatment CT simulation scan with contrast injection on a spiral CT scanner in the treatment position and immobilization. In patients who received induction chemotherapy, CT simulation and MRI after induction chemotherapy were required. In patients not receiving induction chemotherapy, pretreatment CT simulation images were used for target volume delineation; in individuals who received induction chemotherapy, target volumes were delineated in CT simulation images after induction chemotherapy. Scanning scope was from the top of the head to the manubriosternal joint, with 2.5-mm increments. For patients of the PET/CT guided DP-IMRT group (group A), PET/CT scan was performed within 3 days of pretreatment CT simulation scan in the same position and immobilization. The FDG-PET scan was performed 1 h post-injection of 190–240 MBq of FDG. Data were acquired for 3 min per bed position. Patients were well hydrated, fasted for 6 h and glucose levels were below 10 mmol/L. The images were reconstructed to 2 × 2 × 2 mm^3^ voxels using attenuation correction. The FDG-PET images were converted to SUV maps.

In group B, gross tumor volume (GTV) based on CT simulation images was delineated without using the FDG-PET images for dose painting; the corresponding target volumes were derived to achieve an IMRT plan [12–14]. Hard copies of MRI scans, PET/CT images, nasopharyngeal endoscopic examination findings, and pathology reports were available to both groups of patients for consideration in defining the target volumes. The pre-chemotherapy volume of the primary lesion was used for GTVnx (GTV of nasopharynx) delineation. Pre-induction-chemotherapy volumes of involved lymph with extracapsular extension visualized on CT, MRI or PET-CT, and post-induction-chemotherapy volume of involved but encapsulated neck lymph nodes were used for GTVnd (GTV of the lymph node) delineation [14]. In group B, IMRT was delivered using linear accelerators with a nominal energy of 6 MV, and prescribed as follows: GTV of nasopharynx (GTVnx) was expanded outward by 5 mm (including the entire mucous membrane of the nasopharynx and 5 mm below it) [14], defined as PGTVnx, and irradiated at a dose of DT70.4 Gy/32 and DT72.6 Gy/33 Fx in patients with T1-2 and T3-4 disease, respectively, in increments of 2.2 Gy. GTV of the lymph node (GTVnd) was irradiated at a dose of 69.96–72.6Gy/32 to 33 Fx, in 2.12 to 2.2 Gy per fraction. High-risk subclinical lesions (PTV_1_) were irradiated at a dose of DT60.06 to 64 Gy/32 to 33 Fx, in 1.82 to 2.0 Gy per fraction. Lower-risk subclinical disease (PTV_2_) was treated at a dose of DT52.0 to 56.0 Gy/26 to 28 Fx, 1.82 to 2.0 Gy per fraction. Treatment plans were designed and optimized using the Pinnacle inverse planning system. Radiation therapy was administered from Monday to Friday for 32–33 days. In group A, the PET/CT-guided DP-IMRT regimen was used. Target volumes were then delineated on PET/CT images fused with CT simulation images obtained by a group of experienced radiation oncologists, assisted by an experienced nuclear medicine physician. The criteria for defining suspicious disease on FDG-PET/CT relied on visual criteria. Abnormal FDG uptake was defined as an abnormal FDG increased uptake outside normal anatomic structures, higher uptake than background activity or uptake in the location of an anatomic structure, but with asymmetric or higher than expected intensity [11]. Using the simultaneous integrated boost (SIB) technique, a subvolume GTVnx-_PET_ was defined within the GTVnx as the 50% isocontour of the maximum standardized uptake value (SUV50%max). Dose to GTVnx-_PET_ was escalated to DT75.2 Gy/32 Fx in patients with T1-2 disease, and DT 77.55 Gy/ 33 Fx in those with T3-4 disease, in 2.35 Gy per fraction. Other target volumes were delineated as in group B and the same dose was used as in group B. Dose constrains to critical structures were within tolerance according to the RTOG 0225 protocol, and efforts were made to meet these criteria as closely as possible. Median volume of GTVnx was 38.2 ml (range: 5.6–186.6 ml) in group A and 39.4 ml (range: 5.8–176.8 ml) in group B (*P* = 0.42); median volume of GTVnx-_PET_ was 13.8 ml (range: 1.2–32.6 ml).

### Chemotherapy

The induction chemotherapy regimen included TP (docetaxel at 75 mg/m^2^/day or paclitaxel 135–175 mg/m^2^/day on day 1, in combination with cisplatin at 25 mg/m^2^/day on days 1–3), PF (cisplatin at 25 mg/m^2^/day and 5-fluorouracil at 500 mg/m^2^/day on days 1–3). Concurrent chemotherapy was prescribed as 75 mg/m^2^ cisplatin alone every 3 weeks. All patients were scheduled to receive concurrent chemotherapy except for those declining the treatment or presenting severe adverse events. Adjuvant chemotherapy was administered in the same manner as induction chemotherapy. The course of chemotherapy ranged from 3 to 7 cycles.

### Patient assessment and follow-up

Follow-up duration was measured from the first day of treatment to last follow-up date (April 1, 2016) or the date of death. Complete blood cell counts and biochemical profiles were assessed once a week during the treatment period. We graded chemotherapy-related toxic effects in accordance with Common Terminology Criteria for Adverse Events (version 3.0). We graded radiotherapy-related toxic effects in accordance with both the Acute and the Late Radiation Morbidity Scoring Criteria of the Radiation Therapy Oncology Group (RTOG) [1, 9]. Tumor response was evaluated by physical examination, nasopharyngoscopy, and MRI of the head and neck, at 3 months after RT completion. Tumor response was classified according to the WHO response criteria [12, 15]. Complete response (CR) was defined as the complete disappearance of all objective evidence of disease, confirmed by physical examination, direct nasopharyngoscopy, and MRI. After treatment completion, the patients were assessed every 3 months during the first 3 years, and every 6 months thereafter until death or study end. Recurrence was defined as tumor recurrence after the tumor was undetectable for at least 1 month. All local recurrences were diagnosed with fibreoptic endoscopy and biopsy, MRI scan, or both, of the nasopharynx and the skull base showing progressive bone erosion and soft tissue swelling. Regional recurrences were diagnosed by clinical examination of the neck and, in doubtful cases, by fine needle aspiration or an MRI scan of the neck. Distant metastases were diagnosed by clinical symptoms, physical examinations, and imaging methods that included chest radiography, bone scan, MRI, CT, and abdominal sonography.

### Statistical analysis

The primary endpoint was disease free survival (DFS). Secondary endpoints were local failure-free survival (LFFS), regional failure-free survival (RFFS), locoregional failure-free survival (LRFFS), distant metastasis-free survival (DMFS), overall survival (OS), initial response rates after treatment, and toxic effects. LFFS was defined as the time from admission (initiation of treatment) to local failure of primary tumor area. RFFS was defined as the time from admission to failure of non-primary tumor within the treatment regions. Disease free survival was assessed from admission to the date of tumor recurrence, distant metastasis or death, whichever came first. OS was defined as time elapsed from admission to the date of death from any cause or last follow-up. For locoregional and distant failure-free survival analyses, latencies were recorded from admission to first locoregional and remote failure, respectively.

The initial response rates, toxic effects, and other categorical variables were compared by the *χ* ^2^ test. Kaplan-Meier survival curves were used to assess time-to-event endpoints, with the log-rank test used to compare the two groups. Multivariable analyses were performed using the Cox proportional hazards model to evaluate treatment interventions. Potentially important prognostic factors considered in the modeling process were patient age (≤45 vs >45 years), sex (male vs female), tumor stage (T1-2 vs T3-4), node stage (N0-1 vs N2-3), induction chemotherapy (yes vs no), concurrent chemotherapy (yes vs no), pretreatment Epstein-Barr virus deoxyribonucleic acid (EBV DNA) concentration (<4000 copies/ml vs ≥4000 copies/ml) and dose painting (PET/CT-guided DP-IMRT vs CT-based IMRT without DP). All statistical analyses were performed using Statistical Package for the Social Sciences version 18.0 (SPSS, Chicago, IL, USA). All statistical tests were two-sided, with *P* < 0.05 considered statistically significant.

## Results

### Patient characteristics

Between January 2012 and July 2014, 213 patients with locoregional advanced nasopharyngeal carcinoma were included in this study. One hundred and one patients received FDG-PET/CT-guided DP-IMRT, and 112 patients were administered CT-based IMRT. Median follow-up time for all patients was 36 months (range, 8.5–50 months), and 36 months for surviving patients (range, 15.5–50 months). Detailed patient characteristics are shown in Table 1. No significant differences were found between the two treatment groups in terms of baseline demographic and clinical characteristics.

**Table 1:** Characteristics of patients with locoregional advanced NPC assessed in this study

Characteristic	PET/CT-guided DP-IMRT	CT-based IMRT	*p* value*
	No. of patients (%)	No. of patients (%)	
Total	101	112	
Age, y			
Median	46	47	
Range	22–70	18–67	
Sex			
Male	66 (65.3)	78 (69.6)	0.464
Female	35 (34.7)	33 (29.5)	
Pathology			
WHO type 2	30 (29.7)	37 (33.0)	0.659
WHO type 3	71 (70.3)	75 (67.0)	
T stage			
T1	10 (9.9)	13 (11.6)	0.955
T2	27 (26.7)	30 (26.8)	
T3	30 (29.7)	30 (26.8)	
T4	34 (33.7)	39 (34.8)	
N stage			
N0	3 (3)	3 (2.7)	0.654
N1	10 (9.9)	6 (5.4)	
N2	67 (66.3)	79 (70.5)	
N3	21 (20.8)	24 (21.4)	
AJCC stage group			
III	49 (48.5)	59 (52.7)	0.790
IVA	32 (31.7)	31 (30.7)	
IVB	20 (19.8)	22 (19.8)	
EBV DNA			
≥ 4000 copies/ml	49 (48.5)	46 (40.7)	0.334
< 4000 copies/ml	52 (51.5)	66 (59.3)	
Concurrent chemotherapy			
Yes	99 (98.0)	110 (98.2)	0.649
No	2 (2)	2 (1.8)	
Induction chemotherapy			
Yes	26 (25.7)	30 (26.8)	0.875
No	75 (74.3)	82 (73.2)	
Adjuvant chemotherapy			
Yes	8 (7.9)	12 (10.7)	0.490
No	93 (92.1)	98 (89.3)	

*p values were calculated by the *χ* ^2^ test

### Response rates

Complete response rates, evaluated 3 months after RT completion, were 99.0% (100/101) and 92.9% (104/112) in groups A and B, respectively (*P* = 0.037). In group A, one patient had residual neck lymph nodes. Six group B patients had residual neck lymph nodes and two presented residual nasopharyngeal tumors. The two patients with residual nasopharyngeal tumors underwent salvage chemotherapy. Only three patients with residual neck lymph nodes were found in group B at 6 months after RT completion, and successfully underwent salvage neck dissection.

### Adverse events and compliance

All patients in both treatment groups completed the prescribed RT dose. A total of 56 (26.3%) patients received induction chemotherapy. Among them, four patients did not receive concurrent chemotherapy. Concurrent chemotherapy was administered to 209 (98.1%) patients. Adjuvant chemotherapy was provided to 20 (9.4%) patients with tumor presence at the completion of radiotherapy (Table 1). Reasons for withdrawal from chemotherapy included severe mucositis, prolonged severe neutropenia and patient’s refusal. The main grade 3–4 acute adverse events were mucositis and hematologic reactions; grade 3–4 late adverse events included skin fibrosis and ototoxicity (Table 2). No grade 5 toxicity (death) occurred during the treatment. There were no statistically significant differences between the two study groups in cumulative incidence of 3–4 acute and late toxic effects during follow-up (Table 2). One group B patient developed grade 1 temporal lobe necrosis not affecting job performance or daily life activities.

**Table 2:** Grade 3–4 acute and late toxic effects

Adverse events	PET/CT-guided DP-IMRT	CT-based IMRT	*p* value*
	No. of patients (%)	No. of patients (%)	
Acute adverse events			
Anemia	1 (1.0)	2 (1.8)	0.54
Neutropenia	8 (7.9)	11 (9.8)	0.63
Leukopenia	15 (14.9)	18 (16.1)	0.85
Thrombocytopenia	1 (1.0)	1 (0.9)	0.73
Liver dysfunction	0	0	..
Nephrotoxicity	0	0	..
Nausea	14 (13.9)	17 (15.2)	0.85
Vomiting	10 (9.9)	13 (11.6)	0.83
Mucositis	31 (30.7)	34 (32.1)	0.77
Dermatitis	9 (8.9)	12 (10.7)	0.82
Dysphagia or odynophagia	3 (2.9)	5 (4.5)	0.72
Dry mouth	3 (2.9)	6 (5.3)	0.50
Ototoxicity	0	1 (0.9)	0.53
Late adverse events			
Skin fibrosis	0	1 (0.9)	0.53
Dry mouth	0	0	..
Ototoxicity	0	1 (0.9)	0.53
Trismus	0	0	..
Nasopharyngeal ulceration	0	0	..

*p values were calculated by the *χ* ^2^ test

### Patterns of treatment failure

Twelve (11.9%) and 24 (21.4%) patients in groups A and B, respectively, developed tumor recurrence (Table 3). Among the patients who developed distant organ metastases, 26, 11, and 13 had bone, liver, and lung metastases, respectively. Seventeen patients had metastases in more than one organ. Salvage treatments, including chemotherapy, re-irradiation, and surgery, were administered to patients after documented relapse, in accordance with standard practice.

**Table 3:** Incidence and site of recurrence

Site	Incidence, No. (%)	
	PET/CT-guided DP-IMRT	CT-baded IMRT
Locoregional only	3 (3.0)	6 (5.3)
Distant only	8 (7.9)	13 (10.6)
Locoregional and distant	1 (1.0)	5 (4.5)

### Survival

**Fig. 1:**
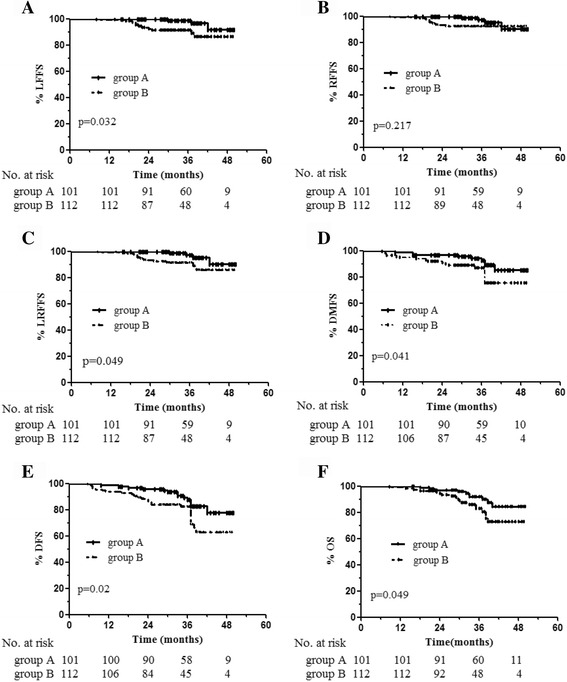
Kaplan-Meier survival curves for the PET/CT-guided DP-IMRT and CT-based IMRT groups. LFFS (**a**), RFFS (**b**), LRFFS (**c**), DMFS (**d**), DFS (**e**) and OS (**f**). *P* values were calculated using the log-rank test

Twenty-nine deaths (10 in group A and 19 in group B) were reported. In group A, causes of death were distant metastases (6 patients), local recurrence and severe malnutrition (2 patients), non-radiation induced cerebral hemorrhage (2 patients). In group B, death was from distant metastases (12 patients), local recurrence and severe malnutrition (5 patients), non-radiation induced cerebral hemorrhage (1 patient) and traumatic brain injury (1 patient). The rates for 3-year LFFS, LRFFS, DMFS, DFS and OS in groups A and B were 98.8% vs. 91.3% (*P* = 0.032), 97.2 vs. 91.2% (*P* = 0.049), 92.9% vs. 87.4% (*P* = 0.041), 87.9% vs. 82.4% (*P* = 0.02), and 91.8% vs. 82.6% (*P* = 0.049), respectively (Fig. 1). No statistically significant differences were observed in RFFS between groups A and B (97.2% vs 92.4%, *P* = 0.217) (Fig. 1). Dose escalation by FDG-PET/CT guided dose-painting IMRT resulted in 7.5%, 6.0%, 5.5%, 5.5% and 9.2% increases in 3-year LFFS, LRFFS, DMFS, DFS and OS rates, respectively, in group A.

### Prognostic factors

The univariate analysis revealed that the factors influencing the 3-year OS were dose painting (PET/CT-guided DP-IMRT vs CT-based IMRT without DP, *P* = 0.049) and EBV DNA concentration (*P* = 0.001), respectively. The factors influencing DFS were dose painting (*P* = 0.020) and EBV DNA concentration (*P* < 0.001), respectively. The factors influencing DMFS were dose painting (*P* = 0.041), EBV DNA concentration (*P* = 0.002), and sex (*P* = 0.026) respectively. The factors influencing LFFS were dose painting (*P* = 0.023) and EBV DNA concentration (P = 0.002), respectively. The factors influencing LRFFS were dose painting (*P* = 0.049) and EBV DNA concentration (*P* = 0.008), respectively. The EBV DNA concentration (*P* = 0.036) was a significant prognostic factor to predict 3-RFFS. However, age, T stage, N stage, induction chemotherapy and concurrent chemotherapy were not the factors for significantly influencing the OS, DFS, LFFS, RFFS or LRFFS. Multivariate analyses showed that dose painting was a significant independent prognostic factor for 3-year LFFS and DFS (*P* = 0.039 and *P* = 0.036, respectively); EBV DNA concentration was a significant independent prognostic factor for 3-year LFFS, DFS, DMFS and OS (*P* = 0.022, *P* < 0.001, P = 0.003 and *P* = 0.003, respectively), and dose painting was of marginal significance in predicting DMFS and OS (*P* = 0.075, *P* = 0.052, respectively). The results of multivariate analysis are summarized in Table 4.

**Table 4:** Multivariable analysis of prognostic factors in locoregional advanced NPC

Endpoint	HR (95% CI)	*p* value*
Local failure-free survival		
Sex	0.608 (0.201–1.844)	0.380
Age	0.955 (0.311–2.937)	0.936
T stage	0.733 (0.197–2.733)	0.644
N stage	0.938 (0.114–7.738)	0.953
Induction chemotherapy	1.321 (0.148–11.759)	0.803
Concurrent chemotherapy	>100 (0.000-..)	0.988
EBV DNA level	0.089 (0.011–0.701)	0.022
Dose painting	0.239 (0.061–0.932)	0.039
Disease free survival		
Sex	1.504 (0.704–3.214)	0.292
Age	1.261 (0.659–2.410)	0.484
T stage	1.504 (0.704–3.214)	0.513
N stage	1.261 (0.629–2.531)	0.649
Induction chemotherapy	1.650 (0.524–5.197)	0.392
Concurrent chemotherapy	0.659 (0.135–3.222)	0.606
EBV DNA level	0.170 (0.069–1.417)	<0.001
Dose painting	0.462 (0.225–0.951)	0.036
Distant metastasis-free survival		
Sex	3.334 (0.991–11.222)	0.052
Age	1.697 (0.781–3.688)	0.182
T stage	1.103 (0.471–2.581)	0.821
N stage	0.688 (0.141–3.354)	0.664
Induction chemotherapy	1.426 (0.298–6.829)	0.657
Concurrent chemotherapy	0.640 (0.072–5.659)	0.688
EBV DNA level	0.218 (0.079–0.601)	0.003
Dose painting	0.453 (0.190–1.082)	0.075
Overall survival		
Sex	1.456 (0.596–3.553)	0.049
Age	1.166 (0.543–2.504)	0.649
T stage	1.264 (0.558–2.860)	0.575
N stage	0.264 (0.032–2.203)	0.218
Induction chemotherapy	2.664 (0.794–8.937)	0.113
Concurrent chemotherapy	0.328 (0.064–1.674)	0.180
EBV DNA level	0.223 (0.082–0.607)	0.003
Dose painting	0.425 (0.179–1.009)	0.052

*p values calculated with the adjusted Cox proportional-hazards model

## Discussion

For locoregional advanced NPC, chemoradiation is the standard of care [1, 12, 16, 17]. Despite recent progress in radiotherapy and chemotherapy, local residual and recurrent disease remains challenging. Dose-escalation in a homogeneous fashion to GTV is, however, restricted by increased risk of toxicity to normal structures (i.e. spinal cord, brain stem and optic nerve). A promising approach to increase local control (and consequently overall survival) is to take advantage of intra-tumor heterogeneity. Local failure in NPC appears most frequently at the primary tumor site and within the irradiated target volume with highest FDG uptake. These findings suggest that boosting these high uptake regions might improve local control. One strategy to accomplish dose-escalation while minimizing toxicity is dose painting, which is defined as locally boosting the tumor to increase locoregional control based on functional imaging (i.e. PET). Dose painting enables mapping of dose prescription to non-uniform distribution of metabolic, biochemical, and molecular abnormalities within the tumor [18–20]. FDG-PET/CT is increasingly used in dose painting for NPC. Integration of FDG-PET in IMRT planning has been described as beneficial for treatment individualization and dose escalation [21]. Furthermore, FDG-PET directed dose distribution could lead to better sparing of organs at risk, such as parotid glands [22, 23]. Various PET-based threshold methods have been proposed for target volume delineation, including SUV2.5, SUV50%max, and signal/background ratio (SBR) [9, 22, 24, 25]. In this study, SUV50%max target was selected as the clinical standard for dose escalation. This choice was motivated by the observation that in ongoing clinical trials, dose painting by contours is performed on SUV50%max isocontour [22, 26]. In general, GTV in IMRT is defined by CT imaging (or fusion of CT with MRI). This study aimed to compare PET/CT-guided DP-IMRT and CT-based IMRT. PET/CT-guided DP-IMRT is an effective technique for dose-escalation, and significantly increases the biologically effective dose (BED) delivered. Based on a linear-quadratic equation with an alpha/beta ratio of 10 for tumor response, the BED was significantly increased to 92.9–95.8 Gy to GTVnx-_PET_ in group A compared to 85.9–88.6 Gy to GTVnx in group B.

Complete response rates after chemoradiotherapy for locoregional advanced NPC vary from 82.8 to 99% [1, 27, 28]. In this study, CR rate was significantly higher in the PET/CT-guided DP-IMRT group than in patients administered CT-based IMRT (99.0% vs 92.9%, *P* = 0.037), suggesting that the risk for local residual tumor was significantly decreased by dose escalation using the DP-IMRT technique. Our findings using a PET/CT-guided DP-IMRT regimen demonstrated that LFFS, DMFS and OS compared favorably to other reports using CT-based IMRT and chemotherapy for NPC patients [14, 28, 29]. Liu et al. [28] reported that 185 patients with stage III to IVb NPC were treated by CT-based IMRT and chemotherapy. For patients with detectable EBV DNA levels and patients with undetectable EBV DNA levels, 3-year LRFS, PFS, and DMFS rates were 82.7% and 93.5%, 71.1% and 85.9%, 86.6% and 90.6%, respectively. He et al. [29] treated 358 patients with locally-advanced NPC using CT-based IMRT with chemotherapy, the 3-year OS, LRFS, DMFS, and DFS were 88.8 vs. 78.4%, 96.5 vs. 91.1%, 87.8 vs. 79.3%, and 84.1 vs. 69.6% for the patients with the distance between the primary tumor and brainstem (Dbs) > 4.7 vs. ≤ 4.7 mm, respectively. Only few studies using DP-IMRT or PET/CT-guided IMRT for NPC have been reported, all with very small sample size. Bakst et al. [10] evaluated 25 NPC patients (stage II-IVB) treated with DP-IMRT and chemotherapy; the prescribed radiation dose was 70.2 Gy using 2.34 Gy fractions to GTV. The 3-year local control rate was 91%, for 91% DMFS and 89% OS. Compared to Bakst et al.’s results, the present study showed better LFFS, DMFS and OS in the PET/CT-guided DP-IMRT group. The reason might be the higher total dose and fractionated dose to GTVnx-_PET_ in the present study than the dose to GTV in Bakst et al.’s study. Wang et al. [9] compared conventional RT (group A), CT-based IMRT (group B) and PET/CT-guided IMRT (group C) in 67 locally advanced NPC patients. For group C, areas with an SUV threshold of 2.5 (SUV2.5) on PET/CT images were defined as GTV, which received 77Gy in group C, in 2.4 Gy per fraction. PET/CT-guided IMRT treated patients showed significantly higher 3-year local progression-free survival (LPFS) and DFS (LPFS, 100% vs 95.8%, *P* < 0.05; DFS, 95.2% vs 79.2%, *P* < 0.05) compared with the conventional RT group. Meanwhile, there was no statistically significant difference in survival rates between PET/CT-guided IMRT and CT-based IMRT groups. The present study showed that 3-year LFFS, LRFFS, DMFS, DFS and OS were significantly higher in PET/CT-guided DP-IMRT group compared with CT-based IMRT group values. A relatively large sample size was assessed here, with the PET/CT-guided dose-painting technique performed on SUV50%max isocontour, which had smaller volumes than in the SUV2.5 based segmentation method [30]. Compared with threshold of SUV2.5, dose escalation on SUV50%max isocontour (GTVnx-_PET_) in this study could potentially improve therapeutic efficacy while reducing the dose to surrounding healthy tissues. Distant metastasis was the major cause of death in patients after treatment; our results suggested that the risk of distant metastasis was significantly decreased with increased local control rates, consequently increasing overall survival.

As shown above, the PET/CT-guided DP-IMRT regimen did not result in increased acute toxicities, indicating that treatment was well tolerated. Furthermore, no patients required significant treatment interruptions during radiation. Late toxicities included skin fibrosis, ototoxicity, and asymptomatic temporal lobe necrosis, and had similar rates between the two groups. Compared to studies by Lin et al. [14] and Lee et al. [12] assessing CT-based IMRT and chemotherapy in NPC patients, the PET/CT-guided DP-IMRT regimen in this study showed no increase in acute and late toxicities. In addition, no in-field cranial nerve injury and massive nasopharyngeal hemorrhage were observed in either group in this study. Wang et al. [9] revealed the most common acute toxicity of PET/CT-guided IMRT to be acute mucositis, while no grade 4 acute toxicities were noted. Late toxicities were subcutaneous fibrosis, xerostomia, and hearing loss. No significant differences were observed in acute and late toxicities between the PET/CT-guided IMRT and CT-based IMRT groups. In this study, limitation of the mean dose to each cochlea was ≤45 Gy, to reduce the incidence of significant hearing loss [13, 31]. One (0.9%) patient with significant tumor extension to the ipsilateral skull base in the CT-based IMRT group developed grade 3 hearing loss. To ensure the radical radiation dose to PTV for tumor control, the ipsilateral cochlea inevitably received a mean dose of 52 Gy. Bakst et al. [10] reported a patient with clinically significant (grade 3) hearing loss. In all, 12% of patients developed temporal lobe necrosis, with one requiring surgical resection. As shown above, only one (0.8%) patient in the CT-based IMRT group developed asymptomatic temporal lobe radiation necrosis 23 months after radiation. This case developed in areas that received the prescription dose of 72.6 Gy because of intracranial tumor extension. The difference in brain toxicities between this study and that of Bakst et al. is likely because the CT-based IMRT group in the current report was treated to a lower fractionated dose. The PET/CT-guided DP-IMRT group presented here generally had smaller GTVnx-_PET_ volumes for dose-escalation, which can explain the low incidence of brain toxicity observed in this work.

A number of studies have reported prognostic factors for NPC patients treated by chemoradiotherapy [1, 14, 28, 29]. EBV DNA is considered an important prognostic factor for NPC patients [28, 32, 33], and previous studies of stratified NPC patients have identified an EBV DNA concentration of 4000 copies/ml as a prognostic cut-off value [32, 33]. As shown above, EBV DNA level was a significant prognostic factor for LFFS, DFS, DMFS and OS. To the best of our knowledge, no previous study has explored the prognostic value of PET/CT-guided DP-IMRT in NPC patients. The current findings revealed dose painting (PET/CT-guided DP-IMRT vs CT-based IMRT without DP) to be a significant independent predictor for LFFS and DFS, with marginal significance in predicting DMFS and OS. However, the therapeutic benefits of PET/CT-guided DP-IMRT regimen should be explored in further prospective studies.

### Limitations

This study was limited by its retrospective nature. Follow-up was not long enough for evaluating long term survival in NPC patients. The major treatment regimen was concurrent chemoradiotherapy with or without adjuvant chemotherapy; however, about a quarter of patients received induction chemotherapy, which may influence treatment homogeneity.

## Conclusion

In summary, our findings indicated that PET/CT-guided DP-IMRT plus chemotherapy is an effective treatment modality for patients with locoregional advanced NPC. This study reiterates that addition of FDG-PET/CT guided dose-painting to IMRT significantly improves survival with no increased toxicities compared with CT-based IMRT in locoregionally advanced NPC. Further prospective randomized studies should be conducted to better define the role of the PET/CT-guided DP-IMRT regimen in chemoradiation for locoregional advanced NPC.

## Abbreviations

BED: Biologically effective dose
CR: Complete response
DFS: Disease free survival
DMFS: Distant metastasis-free survival
DNA: Deoxyribonucleic acid
DP: Dose painting
EBV: Epstein-Barr virus
FDG-PET/CT: ^18^F-fluorodeoxyglucose positron emission tomography/computed tomography
GTV: Gross tumor volume
GTVnd: GTV of the lymph node
GTVnx: GTV of nasopharynx
GTVnx-_PET_: Gross tumor volume of nasopharynx in PET images
IMRT: Intensity modulated radiation therapy
LFFS: Local failure-free survival
LPFS: Local progression-free survival
LRFFS: Ocoregional failure-free survival
NPC: Nasopharyngeal carcinoma
OS: Overall survival
RFFS: Regional failure-free survival
RT: Radiotherapy
RTOG: Radiation Therapy Oncology Group
SBR: Signal/background ratio
SIB: Simultaneous integrated boost
SUV: Standardized uptake value
